# The polar bear in the room: diseases of poverty in the Arctic

**DOI:** 10.3402/ijch.v72i0.21161

**Published:** 2013-08-05

**Authors:** Chris Nelson

**Affiliations:** University of Alaska, Anchorage, USA/University of Oulu, Finland

**Keywords:** Arctic, poverty, circumpolar, disease

## Abstract

In the face of global warming, budgetary austerity and impoverished Arctic residents, the nations of the circumpolar region are presented with a number of difficult choices regarding the provision of health care to the far-flung and isolated regions of their northernmost provinces. Complicating that picture is the reality of neglected tropical diseases in areas far from their perceived normal equatorial range as well as endemic food-borne diseases, including protozoan and helminth parasites, respiratory and gastrointestinal diseases and vaccine-preventable illnesses.

This paper discusses the problems of caring for the health and well-being of indigenous populations suffering from extreme poverty, isolation and discrimination in the circumpolar region. After presenting difficulties as supported by the extant literature, the paper continues by suggesting solutions that include novel telenursing applications, targeted distance-educational programs and local community-based health care assistant (HCA) vocational training. These programs will provide cost-effective care that increases life-spans, improves quality of life and provides opportunities to distressed populations in isolated rural communities of the Far North.

The toolkit presented in the paper is intended to spur discussion on community health programs that could be adopted to provide proper and humane care for marginalized Arctic populations in an extreme and rapidly changing environment.

In any discussion of the difficulties inherent in health care delivery to the Polar region, it is necessary to define the terms. “Polar” encompasses the area near the geographic North Pole while Arctic is traditionally defined as the area north of the Arctic Circle, a circle of latitude found at 66° 33’ 44” north. A more useful working term that has arisen is “circumpolar”, which describes the entire region surrounding the North Pole and is usually expanded to include the areas of land that have an annual high temperature of 10°C. The region encompasses portions of the nations of Sweden, Norway, Finland, Russia, Canada, Iceland and the United States (Alaska), with the inclusion of semi-autonomous regions of Denmark (Greenland and the Faroe Islands).

According to the 2008 United Nations Environment Programme estimate, there are 4,516,700 permanent residents of the circumpolar region, with nearly 10%, an estimated 421,000 residents, considered to be indigenous. The response of various governments to the indigenous people within their borders varies widely with both positive and negative examples. The nomadic Sami have migrations crossing 4 national borders in Fennoscandia and Russia, with rights ranging from full recognition by Sweden and Norway as indigenous peoples under the International Labour Organization's Convention on Indigenous and Tribal Peoples, to Finland acknowledging the Sami as a minority without special rights. Russia chose not to grant special status other than as a “small minority people” with very narrow rights and privileges (that are largely ignored).

The regional distances involved are enormous and create health care access issues for the indigenous populations. For example, in Northern Canada, Young and Chatwood (1) found that “fragmented administrative service and operational services” make health care delivery and continuity an on-going challenge. In addition, “the design and delivery of health services have been oriented mostly along a north-south axis” that channels the health care of residents to southern anchor cities thousands of kilometres away rather than neighbouring communities (1, p. 209). Nurse educators Agnot-Johnston and Ringland found that health care workers in the region “faced [challenging emergent] situations often unimaginable to nurses […] with easier access to physicians, diagnostics, and specialists” and that the health care team “must anticipate the potential for a patient to require an air medical evacuation, factoring in the time and weather conditions for travel” (2, p. 366) across great distances. There is a series of negative impacts on health care when providers struggle with the geography itself while striving to care for their patients: delays in treatment, financial constraints and lack of follow-up visits.

Climate change is strongly implicated in country-food-borne illnesses with the prediction that “higher ambient temperatures in the Arctic may result in an increase in other temperature-sensitive foodborne diseases and influence the incidence of zoonotic infectious diseases by changing the populations and range of animal hosts and insect vectors” (3, p. 22). As an example, the 4 traditional methods of preparing walrus meat are raw, frozen, smoked and as igunaq (buried and left to ferment for a year). None of these methods kill the cold-tolerant trichinosis-causing helminth parasite (*Trichinella nativa*). There have been outbreaks of infection throughout the Arctic regions in Canada and the United States recorded since 1975 (4).

Vitamin deficiencies go hand-in-hand with climate change as well, with changing growing seasons altering traditional gathering patterns and hunting seasons. Low levels of sunlight lead to chronic vitamin D deficiencies and according to Andersen et al. (5), shifting indigenous eating patterns are also leading to a decrease in the intake of vitamin D that was formerly obtained from traditional country foods.

Indoor air quality in Arctic environments is negatively influenced by a number of factors according to Anttonen (6), including pollution, heating system exhaust and close contact necessitated by extreme outdoor temperatures. Respiratory illnesses are endemic, including *B. pertussis*, *S. pneumoniae*, bronchitis, *H. influenza* and *M. tuberculosis*, and the common cold, as well as chronic conditions such as asthma brought about by poor air circulation, allergens, triggers and cold air exacerbation.

Helminth parasitic diseases, including trichinellosis, echinococcosis and diphyllobothriasis, have had periodic outbreaks. For example, “an estimated 40,000 Greenland Inuit were noted to be infected by the mid-1980s out of a population of less than 50,000” (7). Parkinson et al. (3) stress the need for the creation of a strong coalition of International Circumpolar Surveillance in order to identify, isolate and treat infectious disease that extends even further to include *H. pylori*, skin diseases, *S. aureus* (methicillin-resistant), *N. menigitidis* and gastrointestinal rotoviruses.

Another category of illnesses that are of concern are the vaccine-preventable diseases that need a universal childhood inoculation schedule in order to provide both herd immunity and individual resistance. Difficulties in timely delivery of temperature and short-expiration-dated vaccines arise from both the isolated geography and the inclement weather found throughout the circumpolar region (8).

Rural poverty among residents of the far-flung reaches of the circumpolar north is also endemic. In the Arctic “many residents live in small, isolated communities that are dependent on hunting and fishing with little or no economic infrastructure” (3, p. 19). Rural poverty makes the population dependent on traditional country foods, defined as “locally derived plant, animal, and fish foods, which are harvested from the surrounding environment” (9, p. 2). The authors further argue that “decreased intake of country food and increased intake of high-energy, low nutrient market food may put Inuit communities at risk for micronutrient deficiencies, obesity, cardiovascular disease, cancer, and diabetes.” With the effects of climate change, Nancarrow and Chan (9) predict that a “decline in the quality of meat and fish and increase in parasites may have a negative effect on the consumption of nutrients” by the very population that depends on them for food security (p. 9).

Hotez (7, ¶1) makes the argument that poverty is the key to disease spread in the circumpolar region. He states that “the single most important determinant of neglected infections among human populations is the observation that these conditions occur among the poorest people living in the Arctic region.” That the poorest people in the circumpolar region are also the indigenous peoples should come as no surprise, given the lack of economic development and harsh environment in their homelands.

Young and Chatwood (1) argue that even highly developed countries have great difficulties in delivering health care to the isolated regions with outcomes ranging from “Russian regions [that] have low expenditures and poor health status; to the other extreme, such as Nunavut and Alaska … which fail to achieve better health outcomes despite their high level of health expenditures” (p. 211). When the geographic distances are combined with the lack of developed infrastructure, the struggle to provide health care is exacerbated. In Greenland, for example, the vast majority of villages along the coast are unconnected by a road system, necessitating expensive sea and air transport as the only options for transportation.

## Health improvement toolkit: strategies to improve the health of the Arctic poor

An important need in the fight against diseases because of poverty is to address the rights of indigenous peoples in the region. One method is that of partial sovereignty, such as the recognition of Greenland as an Inuit-majority autonomous country within the Kingdom of Denmark (10). This model is also used in the Canadian “First Nations,” which have been granted negotiated rights within their traditional ancestral lands that have led to the creation of self-governed Haida Gwaii and the Inuit-majority province of Nunavut (8). The United States has also held the Alaska Native and American Indian tribal reservations and nations as semi-sovereign, with taxation and legal exemptions from federal law (11).

Varying widely from the semi-autonomy of the Greenlandic, Canadian and US native peoples, the situation in Russia is one of 250,000 members of “linguistic minorities” lumped together without special privileges, such as the Nenets, Khanty and Chuchki. The only exception to the issue of minority rights is in Iceland because alone out of the Arctic nations, it has no indigenous population (12).

Cross-border cooperation is a crucial concern when the nearest provider in a health care emergency could be in another state, province or country. When moving beyond the crisis of medical emergencies, the very similar needs of similar populations in neighbouring areas can lead to coalitions that address shared issues. “Though separated by great distances, [health care workers and researchers] use technology – phone, fax, email – to readily exchange information, supplies and support” (2, p. 365). As a result of the use of more efficient communication technology, groundwork can be laid that promotes improved health outcomes throughout the Arctic region. Immonen et al. (13, p. 842) state that “there is a great need to explore and share knowledge gained through experience within the region about cross-national collaboration.”

Telemedicine is the delivery of patient care from a distance via electronic communications and is 1 component of telehealth, which refers to a broader range of remote health services and information. (14, ¶1).

 Telenursing as defined in this paper is a refined subset of the field that focuses on the provision of care concomitant with Skype visual conferencing for continuity purposes. By building a professional relationship with a specific patient through telenursing, the nurse earns trust that fosters honesty and improved compliance with medicine, exercise, and dietary regimens, regardless of physical distance from the patient's home. Current technological innovations that lead to internet data compression efficiency, multiple provider portals (satellite, fibre optic, landline, cellular, etc.), and intensive investment in internet and cellular infrastructure by regional and national governments, make telenursing strategy a viable option.

Distance education is the second component of the strategy that brings another connection to an isolated settlement that would otherwise lack opportunity to pursue health care options *in situ*. From the Alaska Community Health Aide Program website (15):

The CHAP Distance Learning Network (CHAP DLN) is a method of delivering health care education to the state's most remote villages using the Internet and other technology. The courses are targeted toward those looking to become CHAs for the first time, as well as current CHA/Ps looking to keep their skills current and earn necessary Continue Education units. Courses offered over distance reduce the frequency, length and cost of travel outside of the CHA's village.

With a focus on providing education to both new and current health care workers, the model is much more robust in that all of the connectivity that has been provided becomes informational infrastructure that is an on-going investment in human capital. Economic opportunity is another benefit to the community, as the skills learned through distance education are transferable, valuable, and in short supply/high demand in the circumpolar region.

In-community HCA training is the third plank of this proposed toolkit. There are a number of levels of health care delivery “alternative” professional training, with a wide range of programs available to model. A strong example is Alaska's Community Health Aide/Practitioner program in rural villages, with a focus on training local community members to return to their homes after training to serve as a local health care resource (15). After certification as a Community Health Aide (CHA) and some years of practice, the CHA can return to the program for further education towards becoming a Practitioner (CHA/P), with a much broader scope of practice and responsibility. Similarly, Russia's rural Feldschers fall under the paramedical practitioner category of the International Standard Classification of Occupations (16) and have a level of practice similar to an African Clinical Officer or a United States Physician's Assistant. By providing health care services in rural areas that are difficult to staff with MDs and ARNPs, these various types of “bridge” professionals are invaluable.

## Conclusion

Changing climactic, political and cultural conditions in the circumpolar region are bringing more hurdles to overcome in an already-challenging environment of extreme cold, midnight sun and month-long nights. The extant issues of rural poverty, invasive diseases, and food insecurity when combined with geographic isolation, dangerous weather, and limited resources is a deadly mix that begs for a solution. By coordinating the complimentary tools of telenursing, distance education and local HCA training, the problems can be addressed in a way that invites long-term positive changes in health care outcomes by tackling persistent social and environmental health determinants in a vigorous and empowering way. As a program of greater investment in human capital is made in the Arctic region, more opportunities will arise to intervene in a proactive manner in ways unanticipated by the initial educators. Strengthening the health of communities will have a myriad of benefits both direct and indirect, and coordinated health care action on the part of the governments, NGOs and population active in the region will reap long-term rewards.
